# Mediation Effect of Maladaptive Coping between Work-to-Family Conflict and Cardiovascular Health Behaviors

**DOI:** 10.3390/ijerph192114121

**Published:** 2022-10-29

**Authors:** Hwa-Mi Yang, Hye-Ryoung Kim

**Affiliations:** 1 Department of Nursing, Daejin University, Pocheon-si 11159, Korea; 2 College of Nursing, ShinHan University, 30, Beolmadeul-ro 40beon-gil, Dongducheon-si 11340, Korea

**Keywords:** conflict, coping strategy, health behavior, mediation analysis, stress, psychological

## Abstract

Work-to-family conflicts (WFC), an aggravating factor of stress, may affect cardiovascular health. However, the link between WFC and cardiovascular health behaviors is not fully defined. This study intends to identify the mediating effect of coping strategy on the relation between WFC with cardiovascular health behaviors, such as smoking, stress management, increased physical activity, and healthy diet habits. The study is a cross-sectional online survey with 358 call center counselors. We adopted a linear regression to confirm the relationship between WFC, coping strategy, and cardiovascular health behavior, which are the main variables of the study. Maladaptive coping partially mediated the relationship between WFC and cardiovascular health behavior. Based on the results, we insist that a support system to reduce WFC is necessary, as well as increasing the availability of resources and policies to reduce WFC in the workplace and find ways to improve maladaptive coping to promote healthy behaviors of call center workers.

## 1. Introduction

Cardiovascular disease is one of the most global burdens which need preventive efforts [1]. According to the National Health and Nutrition Examination Survey (NHANES), from 2015 to 2018, the overall prevalence of cardiovascular disease in adults over 20 is 49.2% [2]. Cardiovascular disease, which increases with age, is a significant factor in medical expenses that increase in old age and dramatically affects the quality of life deterioration due to distress [3]. A study reports that work stress causes a 1.1-fold increase in the risk of cardiovascular disease [1]. Thus, stress increases the risk of cardiovascular disease, so stress management is vital to prevent cardiovascular disease. In particular, work stress or work–family conflict may increase the risk of cardiovascular disease, but there is insufficient literature on this [1,4].

Work–family conflict (WFC) is a source of stress experienced by many workers and is a form of role conflict. As people have limited resources and time to perform various roles at work and home, work–family conflict occurs between work and family. This WFC is related to psychological distress, increased turnover, and decreased life satisfaction [5]. Moreover, there are associations between WFC and depression and excessive alcohol use [3]. Some studies have reported that high WFC threatens cardiovascular health [4,6].

According to Lazarus and Folkman’s stress-coping theory, coping is an effort to deal with a demand that exceeds the resources available to an individual or is burdensome [7]. Generally, individuals tend to evaluate harmful/lossy, threatening, and challenging events as stressful, and the emotional and coping responses related to these events may appear [7]. There are various adaptive and maladaptive coping strategies [8]. Appropriate application of various coping strategies affects mental and cardiovascular health [9,10,11].

In a monotonous environment, counselors in call centers face a lot of work stress due to the emotional labor in a one-on-one relationship with many unspecified customers [12]. Such work stress appears as a symptom of emotional exhaustion and adversely affects psychological and physical health [13]. In such a stressful environment, WFC, an aggravating factor of stress, may affect cardiovascular health depending on an individual’s coping strategy [6].

Managing modifiable risk factors such as stress management, increased physical activity, and healthy diet habits can prevent cardiovascular disease. These healthy behaviors can enhance cardiovascular health. However, the link between WFC and cardiovascular health behaviors is not fully defined.

This study intends to identify the behavioral differences according to the WFC level, and the mediating effect of coping strategy on the relation between work–to-family conflicts with cardiovascular health behaviors of call center counselors. We hypothesized as below.

**Hypothesis** **1.** *WFC is negatively associated with cardiovascular health behaviors*.

**Hypothesis** **2.** *WFC is associated with a coping strategy*.

**Hypothesis** **3.** *Coping strategy mediates the association between WFC and cardiovascular health behaviors*.

## 2. Methods

### 2.1. Study Design and Participants

The study is a cross-sectional online survey with 358 call center counselors. Inclusion criteria for the participants were call center counselors who understood the study purpose and agreed to participate, aged 20 to 60.

We calculated the sample size using G-power version 3.1 (Heinrich Heine University, Dusseldorf, Germany). Based on a multiple linear regression analysis of the F-test with the medium effect size of 0.15, the significance level at 0.05, test power at 0.95, and total predictor as 10, the minimum required sample size was 172. However, we included 358 participants for data stability.

### 2.2. Data Collection

We invited 400 call center counselors from an outsourcing service company, which employs most call center staffs in Seoul, South Korea, with more than 30 centers. The outsourcing service company has about 7000 counselors in various industries, including insurance consulting, sales marketing, and customer service. Of the 400 counselors, 358 responded to the invitation.

All participants understood the study’s purpose and procedures and signed informed consent online before the survey. The online data collection period was from 22 November 2021 to 17 December 2021. Duplicated or missing responses were not includded.

### 2.3. Measurements

#### 2.3.1. Dependent Variable: Cardiovascular Health Behaviors

We measured the cardiovascular health behaviors with 18 items on a 5-point Likert scale presented by Korea’s national health insurance service. The scale has six sub-domains of smoking, drinking, exercise, eating habits, stress management, and preventive health care with three items each. The score ranges from a minimum of 18 to a maximum of 90, and the higher the score, the better the health behavior related to cardiovascular disease prevention. The reliability of this scale represented by Cronbach’s ⍺ was 0.70 in the previous study [14] and 0.67 in this study.

#### 2.3.2. Independent Variable: WFC

We measured WFC with the scale by Carson et al. [5]. The WFC scale has nine items on a 5-point Likert scale of 3 items related to time, tension, and behavior, respectively. We calculated the average score of 9 items. A higher score means a higher WFC. The reliability of this tool, represented by Cronbach’s α was 0.86 in a previous study [15] and 0.91 in this study.

#### 2.3.3. Mediator: Coping Strategy

We measured behavioral coping strategies using the Brief COPE Inventory, developed by Carver [16]. It is a 28-item 4-point (ranging from 0 to 3) Likert scale with two subscales classified into adaptive and maladaptive. Adaptive coping is the sum of 16 items (ranging from 0 to 48), and maladaptive coping is the sum of 12 items (ranging from 0 to 36). Higher scores indicate more use of certain types of coping strategies.

Adaptive coping strategies include acceptance, positive reframing, active coping, planning, seeking emotional or instrumental support, Humor, and Religion. Maladaptive coping strategies include self-distraction, venting, substance use, self-blame, denial, and behavioral disengagement. In a previous study, the internal consistency of adaptive and maladaptive coping scales was Cronbach’s alpha 0.88 and 0.81, respectively [17]. In this study, Cronbach’s alpha was 0.83 for adaptive and 0.80 for maladaptive coping.

### 2.4. Statistical Analysis

We analyzed the demographic characteristics of participants with descriptive statistics in terms of frequency, percentage, mean, and standard deviation. We divided WFC high and low groups based on the WFC average score of the participants and analyzed the differences between coping strategies and cardiovascular health behavior through an independent *t*-test.

We adopted linear regression to confirm the relationship between WFC, coping strategy, and cardiovascular health behavior, which are the main variables of the study. We set covariate variables based on *p*-value (<0.20) among participants’ general characteristics, which are associated with cardiovascular health behavior. To verify the mediating effect of the coping strategy in the relationship between WFC and cardiovascular behavior, we adjusted covariates, performed linear multiple regression analysis, and applied Sobel’s test to confirm the mediating effect. We used the SPSS for Windows version 23.0 (IBM, Armonk, NY, USA) for all statistical analyses.

### 2.5. Ethical Considerations

The Institutional review board of Daejin University has approved the ethical considerations in research methods and procedures (IRB number: 1040656-202204-SB-01-02).

## 3. Results

### 3.1. General Characteristics of the Participant

The study participants were call center counselors with an average age of 42.6 years, primarily female, 90.8%, and a regular position employed, 93.9% (Table 1). The working hours per week of the participants was 33.1. The average WFC was 2.8, and maladaptive, adaptive coping strategies were 10.9 and 24.4, respectively. The cardiovascular health behavior score was 54.2 (Table 1).

### 3.2. Behavior Differences according to the Work-to-Family Conflict Level

There were significant differences in maladaptive coping and cardiovascular health behaviors according to the WFC level based on the average score of 2.8 (Table 2). The WFC high group had a higher maladaptive coping score than the low group; among them, the self-distraction score was the highest, followed by venting. Cardiovascular health behavior score was higher in the WFC high group.

### 3.3. Crude Linear Regression Relevant to Cardiovascular Health Behavior

WFC (β = −2.07, *p* < 0.001) and maladaptive coping (β = −5.69, *p* < 0.001) had negative associations with cardiovascular health behavior. On the other hand, Adaptive coping showed a positive association (β = 2.35, *p* = 0.012) (Table 3).

### 3.4. Mediator Effect of Coping in the Association WFC with Cardiovascular Health Behavior

The first equation shows a negative association between WFC and cardiovascular health behavior (β = −1.99, *p* < 0.001). In the second equation, WFC and maladaptive coping showed a positive association (β = 0.16, *p* < 0.001); however, there was no significant association with adaptive coping. WFC continued to affect cardiovascular health behavior directly, although the association was slightly removed after adjusting for maladaptive coping in the third equation (β = −1.42, *p* = 0.007). Maladaptive coping partially mediated the relationship between WFC and cardiovascular health behavior (z = −3.57, *p* < 0.001) (Table 4).

## 4. Discussion

This study investigated mediating effect of copying behavioral strategies between WFC and cardiovascular health behaviors.

We confirmed our first hypothesis of a negative association of WFC with cardiovascular health behaviors through the t-test and the first equation linear regression results. A study reported that women with a high level of WFC are prone to have poor cardiovascular health behavior and a higher incidence of cardiovascular disease [6]. These findings are in line with this study, where the most participants were female.

Even though existing studies are limited to comparing these results directly, some studies have reported the association between WFC and smoking [18], problematic drinking [3,19], and unhealthy eating habit [19]. 

Cardiovascular health behavior is a lifestyle that affects cardiovascular disease and generally includes smoking, drinking, eating habits, exercise, sleep, and stress management. Many studies have tried to understand and improve these health behaviors [20]. However, understanding what leads to unhealthy behavior is insufficient despite the awareness or emphasis on the importance of healthy behavior.

We can consider the relationship between WFC and cardiovascular health behavior from two perspectives.

The first point of view is Lazarus and Folkman’s stress-coping theory. From this point of view, we can put WFC as stress and cardiovascular health behavior as coping. However, in this case, health behavior is limited to unhealthy behavior. According to the view of healthy behavior as a coping strategy for stress, we can understand unhealthy behaviors such as smoking, drinking, overeating, or eating high-calorie foods as maladaptive coping with stress [21]. Stress may directly or indirectly affect health through maladaptive health behaviors [22]. Those exposed to high levels of stress are more likely to have poor health behaviors than those who experience low levels of stress [23,24].

The second perspective is that health behavior is an outcome related to stress. Various coping strategies can be applied to solve stress in stressful situations, but unhealthy behaviors such as overeating, junk food, or smoking are not an essential part of coping but rather a side effect of coping in this perspective. Health behaviors may be affected by stress or coping strategies [21].

Health behavior has a multifaceted aspect with various motives acting on it. Intense stress can negatively affect these motives [21]. We inferred that such a damaged motive leads to unhealthy behavior. In this study, the high WFC group had a lower cardiovascular health behavior score than the low group, suggesting that coping with the high stress was associated with negative health behaviors.

Many studies approach the first point of view, but the second point of view is also necessary to understand health behavior with multifaceted features. The researchers should adopt both perspectives adequately to establish a concept of health behavior, which will be the first step of health promotion.

Our second hypothesis was about the relation of WFC with a coping strategy. A study has reported that the higher stress, the greater the maladaptive coping [25]. Coping is a critical mechanism for mitigating the adverse effects of high stress [26]. In this study, while WFC showed a positive association with maladaptive coping, there was no association with adaptive coping. These results align with a study that reported the mediating role of maladaptive behavioral coping strategies in the relationship between WFC and psychological distress. [11]. According to the study of 429 working women in Malaysia, adaptive coping strategies did not significantly mediate the effects of WFC on psychological distress. However, the maladaptive coping strategy mediates the effect of work–to-family conflict on psychological distress [11]. Furthermore, studies on maladaptive coping for stressful medical conditions report that females use maladaptive coping more than males [27]. The fact that most of the participants in this study were women seems reasonable for the results that more adoption of maladaptive coping strategies to cope with high-stress levels in the WFC high group.

Our third hypothesis was that coping strategy mediates the association between WFC and cardiovascular health behaviors. As a result of the study, we identified the partial mediation effect of maladaptive coping strategies concerning WFC and cardiovascular health behaviors.

The participants in this study used self-distraction and venting the most as maladaptive coping. A study has reported the relation of maladaptive coping strategies, such as self-distraction and venting, to substance use [28].

There are various motives for health behavior [21]. It depends on the person and the situation. For example, people exercise for health, to get in shape, for fun, for social purposes, and to cope with stress. However, it is not easy to do healthy behaviors in highly stressful situations. A study reported the association of high work stress with poor eating and exercise habits [29].

A qualitative study [30] reported the reasons for not being able to resist unhealthy behaviors, even though they are aware that these unhealthy behaviors are harmful to health, as follows. “After a long stressful day, the will to resist unhealthy behavior is exhausted”. In such a stressful situation, thinking is out of control and painful, and maladaptive behavior can prevail.

As such, promoting healthy behavior would be possible through understanding an individual’s various motives, managing stress, and applying appropriate coping strategies.

## 5. Conclusions

In this study, we found that strains of WFC were associated with lower levels of cardiovascular health behavior and that maladaptive coping mediated the relationship between them.

Based on the results of this study, we insist that a support system to reduce WFC is necessary, as well as increase the availability of resources and policies to reduce WFC in the workplace and find ways to improve maladaptive coping to promote healthy behaviors of call center workers.

As a cross-sectional study, there was a limit to establishing a causal relationship, and the fact that most study participants were women requires caution in interpreting the study results. Nevertheless, this study is significant in confirming the mediation effect of maladaptive coping on the relationship between WFC and cardiovascular health behavior.

## Figures and Tables

**Table 1:** General characteristics of the participants (N = 358).

Variables	N (%) or Mean ± SD
Age (year)	42.6 ± 8.29
Gender	
Female	325 (90.8)
Male	33 (9.2)
Education (≥College)	188 (52.5)
Marital status	
Married	179 (50.0)
Unmarried/separated/divorced	179 (50.0)
Number of children	0.9 ± 0.99
Monthly household income (≥4 million KRW)	162 (45.3)
Perceived socioeconomic level (1–5)	2.6 ± 0.64
Years of current work experience	4.4 ± 3.58
Type of employment	
Regular position	336 (93.9)
Non-regular position	22 (6.1)
Work from home	114 (31.8)
Work in the office	244 (68.2)
Working hours per week	33.1 ± 14.83
Work-to-family conflict	2.8 ± 0.72
Maladaptive coping strategies	10.9 ± 5.45
Adaptive coping strategies	24.4 ± 6.83
Cardiovascular health behavior	54.2 ± 7.59

SD = standard deviation; KRW = Korean won.

**Table 2:** Behavior differences according to the work-to-family conflict level.

Variables	Mean ± SD		*t*	*p*
	WFC Low (n = 181)	WFC High (n = 177)		
Maladaptive coping strategies	9.3 ± 4.35	12.6 ± 5.94	−6.01	<0.001
Self-distraction	2.9 ± 1.18	3.4 ± 1.22	−3.67	<0.001
Venting	2.0 ± 1.19	2.6 ± 1.45	−3.92	<0.001
Substance use	1.3 ± 1.66	2.0 ± 2.16	−3.27	0.001
Self-blame	1.2 ± 1.32	1.9 ± 1.54	−4.78	<0.001
Denial	1.0 ± 1.22	1.4 ± 1.43	−3.06	0.002
Behavioral disengagement	0.9 ± 1.16	1.4 ± 1.35	−3.48	0.001
Adaptive coping strategies	24.4 ± 6.51	24.5 ± 7.17	−0.15	0.880
Acceptance	4.1 ± 1.22	4.0 ± 1.30	0.83	0.410
Positive reframing	3.9 ± 1.42	3.6 ± 1.48	2.30	0.022
Active coping	3.7 ± 1.18	3.7 ± 1.29	0.27	0.790
Planning	3.7 ± 1.44	3.6 ± 1.38	1.18	0.241
Seeking instrumental support	2.7 ± 1.31	2.9 ± 1.37	−1.50	0.133
Humor	2.6 ± 1.50	2.7 ± 1.51	−0.83	0.409
Seeking emotional support	2.1 ± 1.36	2.6 ± 1.54	−3.69	<0.001
Religion	1.5 ± 1.69	1.4 ± 1.69	0.73	0.467
Cardiovascular health behavior	55.1 ± 7.85	53.2 ± 7.21	2.29	0.022

SD = standard deviation; t = independent t-test; WFC = work-to-family conflict.

**Table 3:** Unadjusted associations of cardiovascular health behavior (N = 358).

Variables	β (SE)	*p*
Age (year)	0.32 (0.045)	<0.001
Gender (men)	−6.10 (1.350)	<0.001
Education (≥some college)	1.68 (0.799)	0.036
Marital status (married)	1.25 (0.800)	0.119
Number of children	1.18 (0.400)	0.003
Monthly household income (≥4million KRW)	1.14 (0.803)	0.073
Perceived socioeconomic level (1–5)	1.20 (0.625)	0.056
Years of current work experience	0.21 (0.112)	0.060
Type of employment (regular position)	0.38 (1.672)	0.821
Work from home	−0.14 (0.862)	0.873
Working hours per week (≥40hous)	0.81 (0.878)	0.358
Work-to-family conflict	−2.07 (0.544)	<0.001
Maladaptive coping strategies	−5.69 (0.833)	<0.001
Adaptive coping strategies	2.35 (0.933)	0.012

β = unstandardized regression coefficient; SE = standard error.

**Table 4:** Mediation analysis of coping strategies between WFC and cardiovascular health behavior.

Variables	β (SE)	*p*
Maladaptive coping		
First equation		
Outcome variable: Cardiovascular health behavior		
Independent variable: WFC	−1.99 (0.520)	<0.001
Second equation		
Outcome variable: Maladaptive coping		
Independent variable: WFC	0.16 (0.031)	<0.001
Third equation		
Outcome variable: Cardiovascular health behavior		
Mediator: Maladaptive coping	−3.74 (0.876)	<.001
Independent variable: WFC	−1.42 (0.525)	0.007
Sobel’s test, z = −3.57, *p* < 0.001		
Adaptive coping		
First equation		
Outcome variable: Cardiovascular health behavior		
Independent variable: WFC	−1.99 (0.520)	<0.001
Second equation		
Outcome variable: Adaptive coping		
Independent variable: WFC	−0.01 (0.032)	0.985
Third equation		
Outcome variable: Cardiovascular health behavior		
Mediator: Adaptive coping	1.87 (0.862)	0.031
Independent variable: WFC	−1.99 (0.517)	<0.001
Sobel’s test, z = −3.29, *p* < 0.001		

β = unstandardized regression coefficient; SE= standard error; WFC= work–family conflict. Multiple regression models adjusted for age, gender, education level, marital status, number of children, monthly household income, perceived socioeconomic level, and years of work experience.

## Data Availability

The data supporting this study’s findings are available from the corresponding author (H.-R.K.), upon reasonable request.
